# The Influence of Three-Dimensionally Printed Polymer Materials as Trusses and Shell Structures on the Mechanical Properties and Load-Bearing Capacity of Reinforced Concrete

**DOI:** 10.3390/ma17143413

**Published:** 2024-07-10

**Authors:** Mohammad Hematibahar, Ali Hasanzadeh, Makhmud Kharun, Alexey N. Beskopylny, Sergey A. Stel’makh, Evgenii M. Shcherban’

**Affiliations:** 1 Department of Reinforced Concrete and Stone Structures, Moscow State University of Civil Engineering, 26 Yaroslavskoye Highway, 129337 Moscow, Russia; miharun@yandex.ru; 2 Department of Geotechnical Engineering, Babol Noshirvani University of Technology, P.O. Box 484, Babol 4714871167, Iran; a_hasanzade64@yahoo.com; 3 Department of Transport Systems, Faculty of Roads and Transport Systems, Don State Technical University, 344003 Rostov-on-Don, Russia; 4 Department of Unique Buildings and Constructions Engineering, Don State Technical University, 344003 Rostov-on-Don, Russia; sergej.stelmax@mail.ru; 5 Department of Engineering Geometry and Computer Graphics, Don State Technical University, 344003 Rostov-on-Don, Russia; au-geen@mail.ru

**Keywords:** 3D printing, polymers, high-performance concrete, ultra-high-performance concrete, hyperboloid, shell structure, polymer reinforcement

## Abstract

Three-dimensional printing technology (3D printing) is becoming a more and more popular technology for aerospace, biology, medicine, mechanics, civil and other engineering fields. In civil engineering, researchers and engineers attempt to print construction materials such as concrete using 3D-printing technology. This study aims to investigate the reinforcement of concrete beams with 3D printing. To achieve this, fused deposition modeling (FDM) technology as a printing method and polylactic acid (PLA) as a material were selected. Two types of geometries were chosen to find the optimal mechanical behavior of concrete: high-performance concrete (HPC) reinforced with four types of trusses (Pratt, Howe, Warren, and Warren with vertical) and ultra-high-performance concrete (UHPC) reinforced with a hyperboloid shell structure. The compressive and tensile strengths of reinforced UHPC were examined by a three-point bending test, and reinforced HPC was examined by a four-point bending test. The results of the experiments show that hyperboloid shell structures can absorb energy, although the strength of reinforced UHPC is reduced. For example, there was a decrease of over 20% in the compressive strength and 41% in the flexural strength, but the ductility was raised. Adding the hyperboloid shell structure improved the deformability of the UHPC. When Warren and Howe trusses were added to the HPC as reinforcements, the flexural strength improved by over 26% and 4.3%, respectively. The overall results of this study show that the concrete reinforced with 3D-printed trusses was better than that with a hyperboloid shell structure.

## 1. Introduction

Many researchers have attempted numerous studies to improve the mechanical properties of concrete and cement matrixes with reinforcements using 3D-printing technology [1,2,3]. Therefore, additive manufacturing to strengthen cementitious materials has become an important topic in civil and materials engineering. Researchers try to improve the mechanical properties and bearing capacity via different geometries of 3D-printed structures. For example, Zou et al. [4] offered cementitious tailings backfill (CBT) reinforced with 3D-printed polymers. According to their report, the flexural strength of CBT reinforced with 3D-printed polymers was improved by more than 409%. In another example, when a 3D-printed polymer in the form of a cross was added to CBT, the tensile strength improved by more than 31.6% [5].

Additive manufacturing has many beneficial effects on reinforced concrete and cement mortar. For example, Hack et al. [6] used glass fiber fabricated via three-dimensional printing to make fiber-reinforced polymers (FRPs) to reinforce the concrete and cement mortar. Another example is the use of a 3D-printed fiber and PLA as reinforcing materials for concrete and cement mortar due to its improved acoustic properties. According to the results, PLA cannot absorb a high amount of sound compared to cotton fiber [7]. Farina et al. [8] added 3D-printed PLA to cement mortar to reinforce a cementitious beam. Their results showed that when a 3D-printed rebar was used with a smooth surface, the load–deflection behavior displayed strain softening, while when 3D-printed rebar was used with a lateral surface, the load–deflection behavior changed to displaying strain hardening.

According to the literature, different studies have analyzed different 3D-printed materials. Tzortzinis et al. [9] studied the effect of a geometrically printed hexagon auxetic lattice reinforced with steel materials. They found that the compressive strength of the reinforced material improved by over 140% and the compressive strain increased by over 20%. Another study used two different patterns (honeycomb and triangle) of reinforced cementitious materials through fused deposition modeling (FDM) with polyethylene terephthalate glycol (PETG), PLA, and acrylonitrile butadiene styrene (ABS) as the printing materials. According to their results, when the honeycomb pattern was used as a reinforcement in a cement material, the flexural strength and capacity of deflection increased by over 46.8% and 251.85%, respectively. In addition, unlike using the triangle pattern, adding honeycomb as a reinforcement pattern to cementitious materials can change the strain softening of the cement material beam to strain hardening [10]. Katzer and Szatkiewicz [11] analyzed similar research to investigate strain hardening. They reinforced a cement mortar 3D-printing-reinforced beam (160 × 40 × 40 mm^3^) to achieve strain hardening. To achieve this, they printed geometric honeycomb cells with different thicknesses. They found that the best thickness for improving the strain hardening was 20 mm.

In another method of using 3D-printing technology to improve the mechanical properties of cement material and concrete, Xu et al. [12] used a cellular mold with an auxetic geometry to cast the cementitious material and investigated it under cyclic loading. They reported that 2.5% reversible deformation was obtained when the cycle loading reached 25,000. In another experiment, Aghdasi et al. [13] used a polymer thermoplastic to print a geometric octet truss shape and filled the inside with ultra-high-performance concrete (UHPC). According to their analysis, the octet truss’s flexural strength was more than that of a conventional beam. Auxetic materials are known as negative Poisson ratio materials [14]. The first negative Poisson ratio material made, in 1987 by Lakes, was an auxetic foam structure [15,16,17]. A negative Poisson ratio provides favorable mechanical properties such as impact resistance, ultra-high stiffness, zero shear modulus, and fracture toughness because of the geometrical shape patterns of acoustic structures [18,19]. At present, cellular structures display behavior like that of a honeycomb structure with a negative Poisson ratio [20]. Nowadays, many researchers are attempting to study the effect of auxetic materials on cement and concrete. For example, Fan et al. [21] added auxetic foams as fibers to a cementitious matrix at different volume fractions (0.5–2%). Their results demonstrate that the addition of auxetic foam fibers decreases the mechanical properties of the cementitious material but enhances the toughness of the mortar. Askarinejad et al. [22] investigated the tensile strength of mortar with different auxetic geometries. The authors found that the best tensile strength results were for the super wave auxetic geometry, with an increase of over 42%. In another example, Xu et al. [23] analyzed a cement material reinforced with an auxetic hyper-elastic frame. They attempted to analyze this auxetic hyper-elastic frame under cyclic loading. According to their results, the strain of this reinforcement type increased by over 40%, and deformability improved by over 10%. In another example, Xu and Savija [24] analyzed the effect of a different pattern of an auxetic 3D-printing-reinforced cement material. Results of 3D-printed re-entrant (RE) auxetic material to reinforce the cement matrix were 853% and 708% for ductility and energy absorption, respectively. Although some studies reach the optimal flexural strength of a reinforced concrete beam through 3D-printing-reinforced technology and find the strain-hardening of the concrete beam, there is no simple design or method for strengthening through 3D-printing technology. Three-dimensional printing-reinforced cement is a wide area for improving civil and material engineering.

Many studies understand the positive effect of 3D-printing reinforcement on cement mortar such as ductility and high displacement. In addition, three-dimensional printing reinforcement has a negative effect too. To highlight the positive effects of incorporating 3D printing into cement mortar, Xu et al. provided an example [23] that used auxetic cementitious composite (ACC). It shows that the strain hardening improved by over 40%. Moreover, they understood that the auxetic structure can improve stiffness/strength and energy dissipation plateau under cyclic loading. In another example, when different types of ACC were added to cement mortar, the mechanical properties were improved. For example, when re-entrant (RE) was added to cement, the ductility was improved by over 853%, and energy absorption was increased by over 703%, while rotating squares (RSs) increased compressive strength by over 18% [24]. A negative effect of adding 3D-printed elements to cement mortar is the deterioration of deformation characteristics. For example, Xu et al. [2] studied a 3D-printed polymer grating in cement slurry. The authors showed that when lattice trusses are added to the cement mortar, the deformation behavior changes to soften the deformation. Thus, it can be seen that adding 3D-printed elements to cement mortar has both positive and negative effects.

The analysis of literature sources shows a gap in the scientific field regarding the optimal form and application of polymer reinforcement. This study mainly focused on the influence of four types of polymer trusses (Pratt, Howe, Warren and Warren with vertical) and hyperboloid shell structure to find the best concrete performance enhancer. In this study, reinforcements were printed using FDM technology with PLA as the printing material. The trusses used were made of HPC and a hyperboloid shell structure reinforced with UHPC.

## 2. Materials and Methods

**Figure 1:**
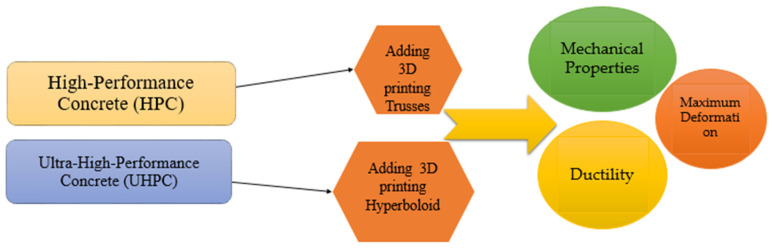
The development of 3D-printing-reinforced concrete.

The results of our previous extensive studies and the experiments by Hematibahar et al. [25] and Chiadighikaobi et al. [26] formed the basis for the present study. Therefore, the application of a hyperboloid and four types of trusses was investigated to understand the differences between the mechanical properties of 3D-printing-reinforced concrete beams. Figure 1 shows the similarities and differences between 3D-printed hyperboloid- and truss-reinforced concrete.

In the first experiment, high-performance concrete reinforced with truss structures was investigated, and in the second experiment, ultra-high-performance concrete reinforced with hyperboloid structures was studied.

It should be noted that the trusses and hyperboloid structures reinforced HPC and UHPC beams, respectively.

### 2.1. Three-Dimensional Printing Materials and Fabrication

Both 3D-printed structures were made from PLA materials. Table 1 shows details of the production differences between the hyperboloid and trusses. The trusses and hyperboloid structures were produced by 3D printing with the same layer thickness of 20 μm, printing speed of 50 mm/s, extruder temperature of 190 °C and layer temperature of 60 °C. According to Table 1, the 3D-printed trusses and the 3D-printed hyperboloid structure were 45% and 30% filled, respectively. Although infill percentages were different, geometry is more important than the infill percentage.

PLA was obtained from (*C*_3_*H*_4_*O*_2_)_*n*_ as the backbone formula that includes lactic acid with water loss. PLA is an environmentally friendly material used in different industries with many printable properties [27,28].

**Figure 2:**
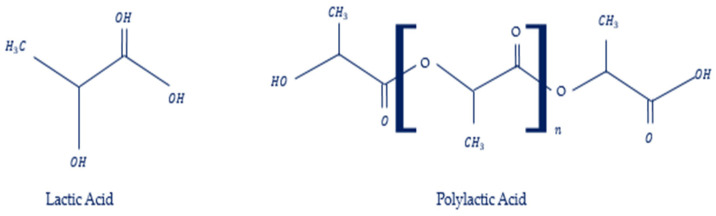
Lattice and polylactic acid formula.

Lactic acid (LA) was discovered as an essential process of the glycolytic energy cycle of living organisms in 1881 through the extraction of fermented milk [29,30]. The synthesis of PLA is a multistep process that starts with the production of LA and ends with polymerization when lactide formation acts as an intermediate step [31]. Finally, the PLA chemical component chain is prepared for Figure 2. Recently, many studies have investigated the combination of PLA and PLA products to find optimal materials for 3D-printed filaments [32,33].

**Figure 3:**
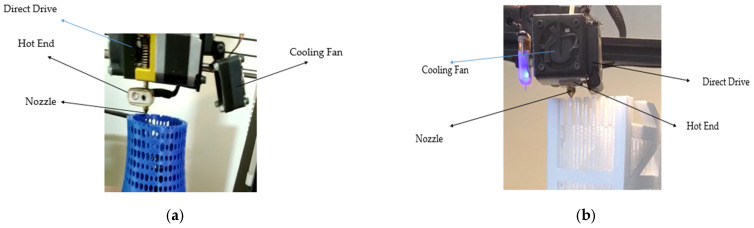
The direct drive, hot end and nozzle: (**a**) UHPC reinforced with hyperboloid shell structure; (**b**) HPC reinforced with trusses.

The fabrication process was based on the FDM method. In this method, the filament was dissolved by the hot end of the FDM printer and extruded through the nozzle. Both 3D-printed forms were fabricated by Direct Drive, extruding the fabrication method. In to this method, a motor and gears push filaments towards hot blocks, and the nozzle extrudes the dissolved filaments. Studies show that most fabrication methods are based on direct drive. There are two common three-dimensional printing fabrication methods (Cartesian and Delta). Cartesian printers can move its joints and arms, while Delta printers can move in a special triangular shape in XYZ direction. Another advantage of the Cartesian model is the ability to print in XY (flat), XZ (edge), and YZ (straight) directions [34,35,36]. Both investigations used a Cartesian 3D-printer model to print reinforcements for concrete beams. Figure 3 illustrates the direct drive, nozzle and hot end of both 3D printers.

**Figure 4:**
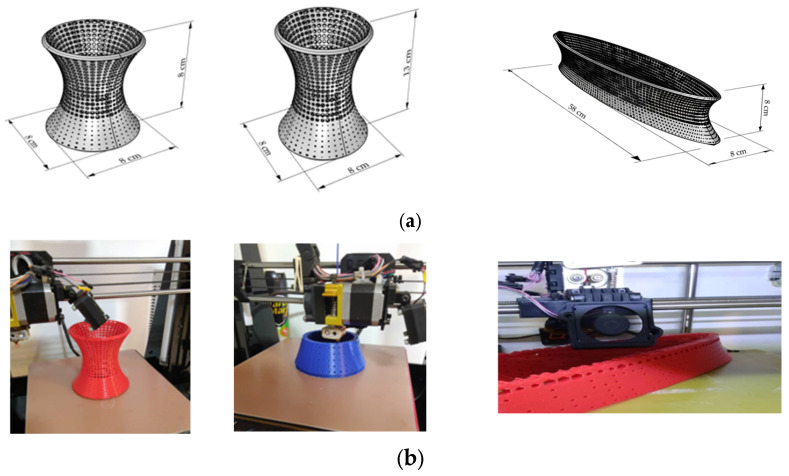

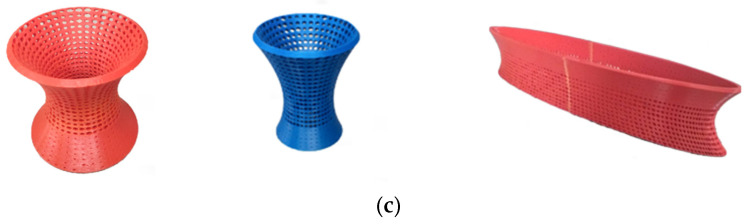
Computer modeling, fabrication and complete 3D printing: (**a**) hyperboloid forms, (**b**) 3D printing, (**c**) prototypes.

To determine the reinforced concrete mechanical properties with a hyperboloid structure, 3D-printer-fabricated hyperboloids were prepared as follows: a 100 mm × 100 mm × 100 mm compression cube, 100 mm diameter and 200 mm height tensile cylinder, and 600 mm × 100 mm × 100 mm flexural prism. Figure 4 illustrates the 3D-printing dimension, fabrication and complete printing of the hyperboloid structures.

The hyperboloid structures included two double curvatures with opposite directions and high resistance to buckling. Hyperboloid structures provide many mechanical properties with fewer materials and are very economical [37]. This type of structure can improve load-carrying capacity to change the fracture structure mode [38,39]. Hexagonal honeycomb has higher stiffness impact resistance and higher energy-absorbent capacity [40,41,42,43,44]. In addition, honeycomb structures are lightweight and have the best performance. According to the investigation, the thickness of a layer of honeycomb structure isrelated to the mechanical properties of the structure [45,46,47,48,49,50,51]. This investigation used hyperboloid structures with honeycomb cells to find the best performance and identify the maximum absorbent energy of reinforced concrete.

Another structure used to reinforce concrete is trusses. Four types of trusses wereadded to concrete to find the best truss type. In this investigation, Pratt, Howe, Warren, and Warren with vertical trusses were added as reinforcements to concrete.

Pratt trusses comprise triangular membranes and diagonal elements. Research has proven that diagonal elements are under tension and vertical elements are under compression during loading [52]. Howe trusses are similar to Pratt trusses, with diagonal elements that slope towards into center [53]. Warren trusses consist of equilateral triangles and diagonal members, while Warren trusses with vertical are similar to Warren trusses but with vertical members added.

**Figure 5:**
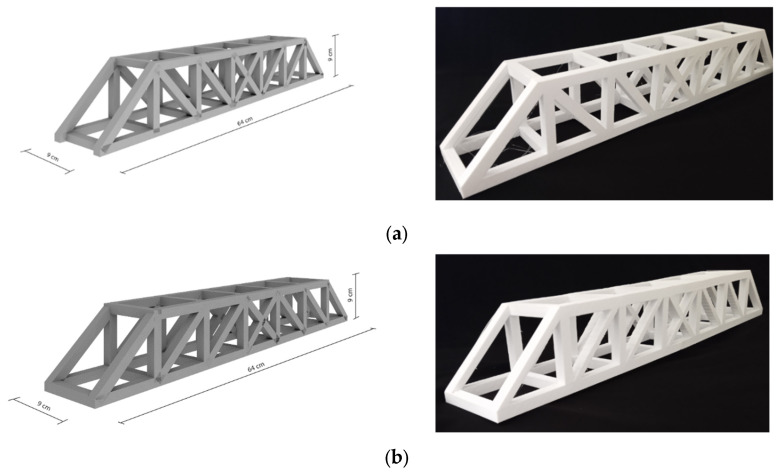

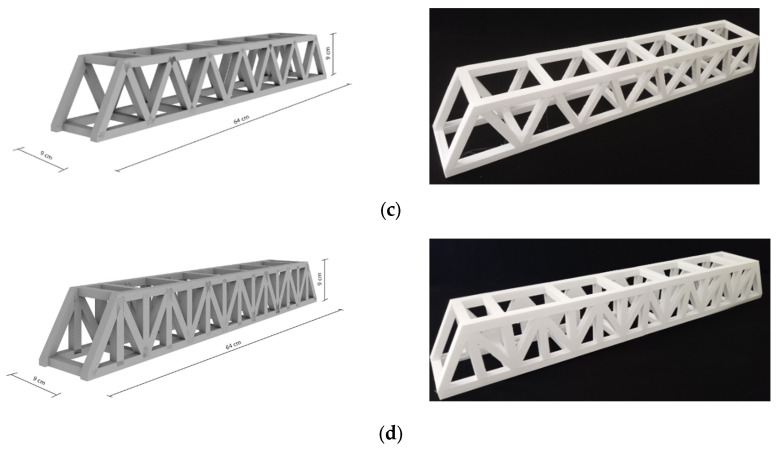
Computer modeling and complete 3D printing; (**a**) Pratt truss, (**b**) Pratt truss, (**c**) Warren truss, (**d**) Warren with vertical truss.

**Figure 6:**
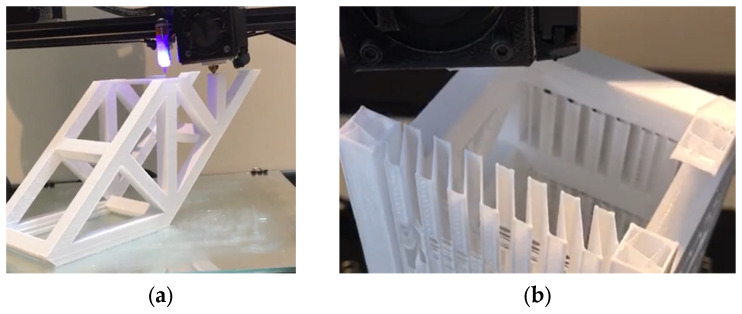
Fabrication method of trusses: (**a**) printing process; (**b**) truss cross-section.

Figure 5 shows the four types of trusses used as three-dimensional-printed reinforcements for concrete. Figure 6 illustrates the dimensions, fabrication and complete 3D printing of four types of trusses.

The PLA material parameters are shown in Table 2. According to Table 2, the ultimate tensile strength was over 57.16 MPa, the yielding strength was over 52.47 MPa, and the maximum strain was over 2.35%.

### 2.2. Concrete Materials

Two types of concrete were selected for reinforcement with three-dimensionally printed PLA materials: HPC concrete reinforced using four types of 3D-printed trusses, and UHPC concrete reinforced using a 3D-printed hyperboloid shell.

**Figure 7:**
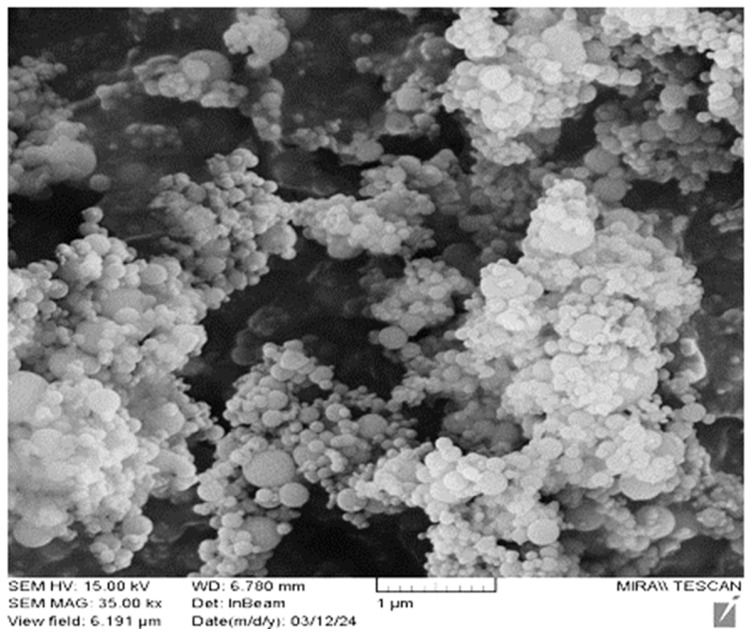
The amorphous shape of silica fume in scanning electron microscope (SEM).

The HPC design used a mixture of ordinary Portland cement (OPC), sand (as fine aggregates), gravel (as coarse aggregates), tap water, super-plasticizer and silica fume. The chemical compositions of OPC and silica fume are shown in Table 3. It should be noted that the silica fume was produced from silicon and ferrosilicon. In this process, amorphous silicon (SiO_2_) with a diameter of 30 nm and 300 nm was separated from gaseous silicon oxide (SiO) when the silicon and ferrosilicon of electric arc furnaces were immersed. In general, the diameter of silica soot is not more than 100 nm. One of the important features of silica fume is its reaction with calcium hydroxide and the formation of calcium hydro silicate, which is called (C-S-H), during hydration [55,56,57]. Figure 7 illustrates amorphous silica fume in a scanning electron microscope (SEM).

**Figure 8:**
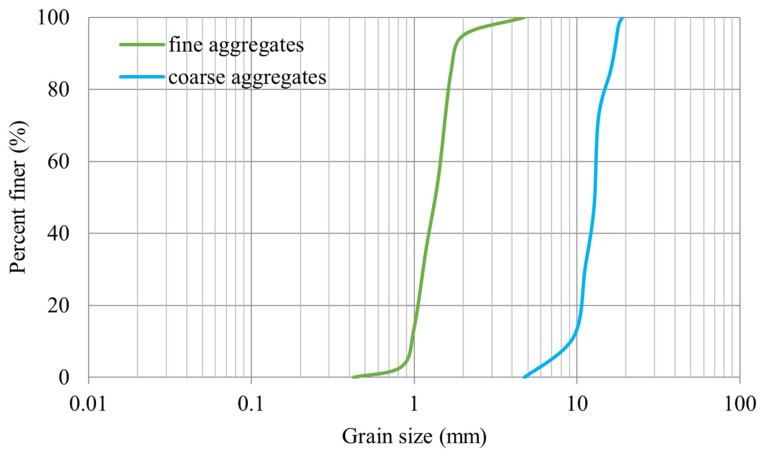
Gradation curves of sand and gravel used for HPC.

Table 4 demonstrates the mixture composition of HPC. According to the HPC mixture composition, the water to cement ratio was 0.375, water to super-plasticizer ratio was 0.06, and minimum and maximum aggregate size of sand and gravel were 0.2 and 2 mm to 7 to 20 mm, respectively. The grain size distributions for fine and coarse aggregates are shown in Figure 8.

**Figure 9:**
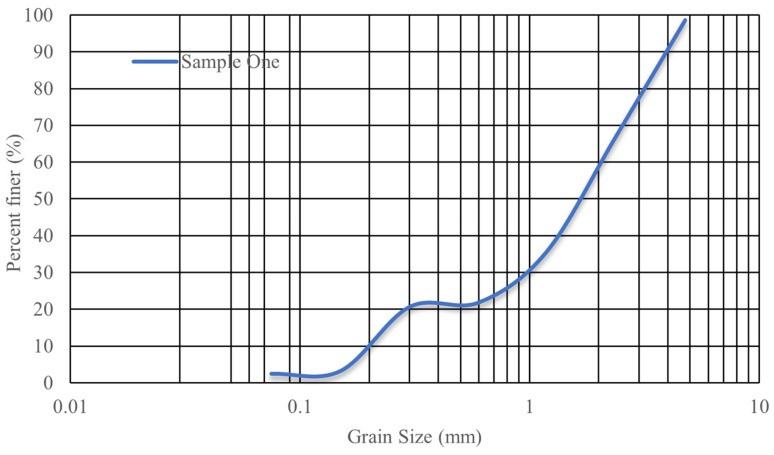
Gradation curves of fine aggregates for UHPC.

UHPC concrete reinforced with a hyperboloid shell structure was designed with cement, water, superplasticizer, sand, gravel and silica fume. The mixture design of UHPC is shown in Table 5. The water–cement ratio was 0.25, and the water to super-plasticizer ratio was 0.06. As shown in Figure 9, the maximum fine aggregate dimension was 2.36 mm, and the minimum was 0.075 mm.

### 2.3. Experimental Method

#### 2.3.1. HPC Reinforced with Trusses

**Figure 10:**
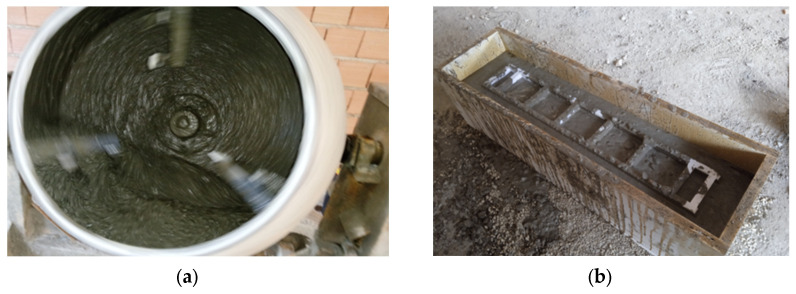
The cast of HPC: (**a**) pan mixer; (**b**) casting concrete in mold.

**Figure 11:**
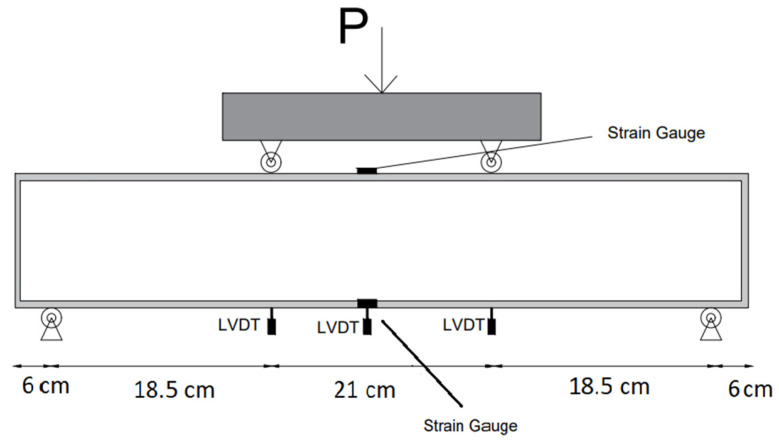
Scheme of the four-point bending test.

First, HPC was mixed in a concrete pan mixer; second, a three-dimensional form was placed in the mold, and then the concrete mixture was poured into the mold (Figure 10). The HPC reinforced with 3D-printed trusses was examined by a flexural prism for reinforced concrete (four-point bending test), compressive cube, and tensile cylinder. Figure 11 illustrates the scheme of the four-point bending test.

#### 2.3.2. UHPC Reinforced with Hyperboloid

**Figure 12:**
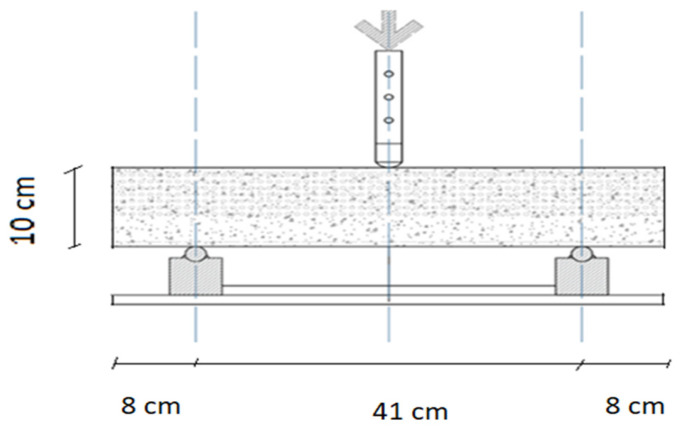
The scheme of the three-point bending test.

**Figure 13:**
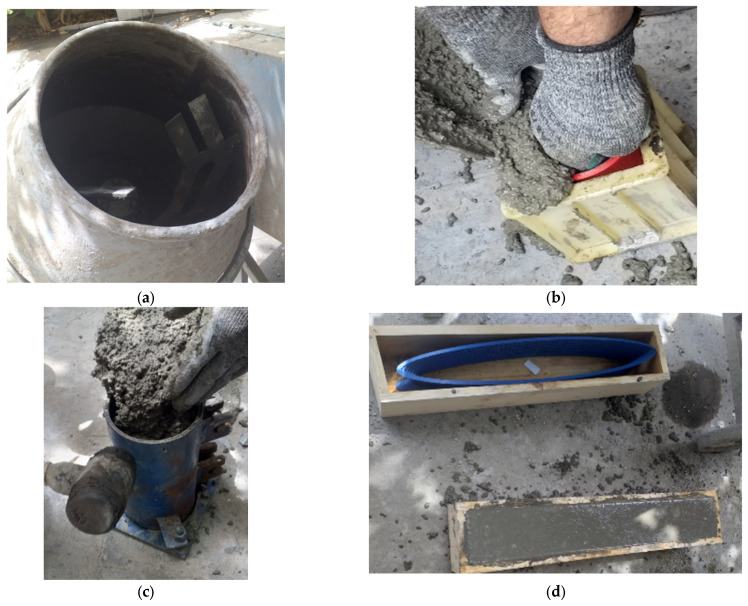
The cast of UHPC: (**a**) pan mixer; (**b**–**d**) casting concrete in molds.

The cubes for compression test, cylindrical samples for tension, and prism for flexural test were reinforced with a hyperboloid shell. Figure 12 shows the flexural test scheme, which follows the three-point bending method. According to the process, first, UHPC concrete was mixed in a pan mixer, and a hyperboloid shell was placed into the mold. Next, the concrete samples were installed in formworks (Figure 13).

Based on GOST 10180 and ASTM C109 [60,61], a compressive strength test was conducted. Tensile strength was determined under ASTM C496 [62]. The flexural strengths determined in four-point bending and three point bending tests were based on ASTM C1609 and ASTM C293 [63,64].

## 3. Results

### 3.1. HPC Reinforced with Trusses

**Figure 14:**
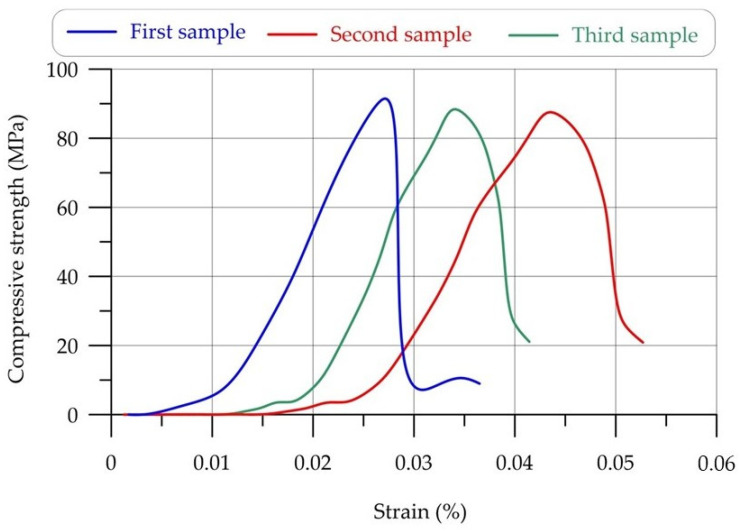
Compressive stress–strain diagram of HPC.

The compressive strength of HPC material was over 87.6 ± 1.75 for three replication samples. Figure 14 illustrates the stress–strain curve of the compressive strength of HPC. Figure 14 shows that the ultimate deformation of the structure varied, although the compressive strength was almost constant. The dissimilarity arises from the application of disparate reinforcement strategies.

Four-point bending testing of truss-reinforced HPC produced mixed results. The flexural strength results for each HPC-strengthened truss were checked one by one.

**Figure 15:**
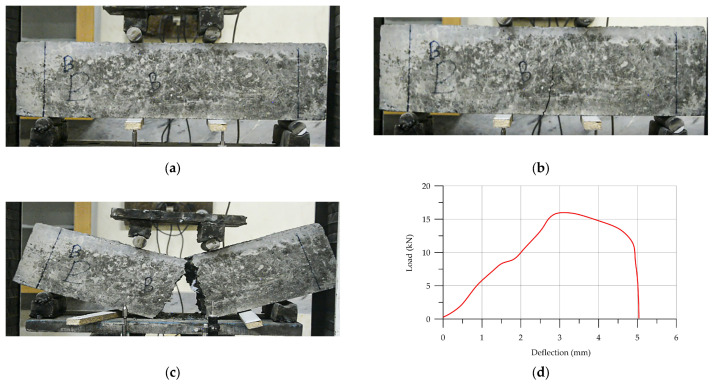
The load–deflection of (P-HPC): (**a**) initial sample of P-HPC prism; (**b**) crack; (**c**) failure; (**d**) load–deflection diagram.

For the first sample, 3D-printed Pratt-reinforced HPC (P-HPC) was analyzed to find the best reinforced shape. Figure 15 shows a load–deflection diagram and failure of 3D-printed Pratt-reinforced HPC. Once the flexural load reached 15.9 kN and the deflection reached 3.04 mm, the first crack appeared. The cracking progress is shown in Figure 15a. The failure of the specimen occurred gradually as the cracking progress advanced. It is important to mention that the failure process was not sudden or severe. Table 6 illustrates the results of the load–deflection diagram results for each specimen.

**Figure 16:**
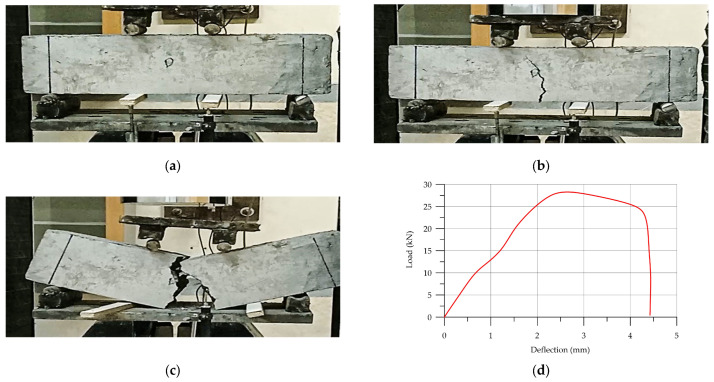
The load–deflection diagram of (H-HPC): (**a**) initial sample of H-HPC prism; (**b**) crack; (**c**) failure; (**d**) load–deflection diagram.

3D-printed Howe-reinforced HPC (H-HPC) failure results and load–deflection diagram are depicted in Figure 16. According to the load–deflection diagram and failure, the cracking progress started when the flexural strength was 25.5 kN and deflection was 3.19 mm. According to Figure 16a,b, the cracking progress was not sudden or harsh. Table 7 shows the details of the flexural strength results.

**Figure 17:**
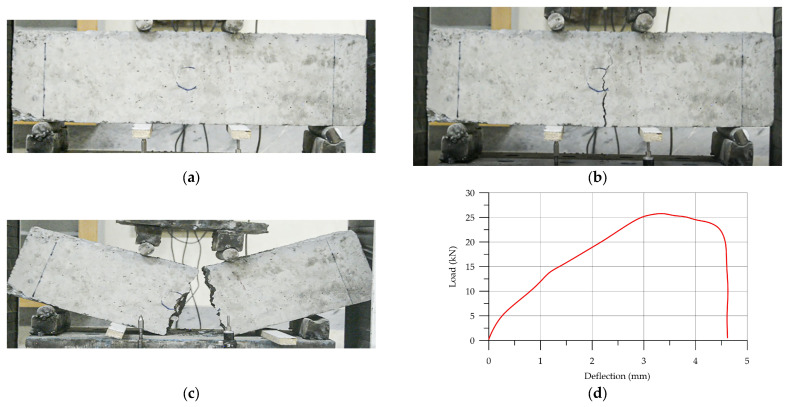
The load–deflection of W-HPC: (**a**) initial sample of W-HPC prism; (**b**) crack; (**c**) failure; (**d**) load–deflection diagram.

Failure and load–deflection diagram of 3D-printed Warren-reinforced HPC (W-HPC) are shown in Figure 17. Like other types of 3D-printing-reinforced concrete, adding a Warren truss to reinforce HPC had a similar effect of improving the maximum load of the HPC. The crack progression of W-HPC exhibited sudden cracking, slow crack propagation, and eventual specimen failure. Figure 17a illustrates the cracking process of the W-HPC sample. Table 8 shows the load–deflection results.

The failure and load–deflection curve of W-HPC are shown in Figure 17. Like other types of 3D-printing-reinforced concrete, the application of a Warren truss to reinforce HPC had a similar effect of improving the maximum load capacity of the HPC. Cracking in the W-HPC was revealed suddenly. The crack grew and progressed slowly, eventually leading to the failure of the specimen. Figure 17a illustrates the cracking process of the W-HPC sample. Table 8 shows the load–deflection results.

**Figure 18:**
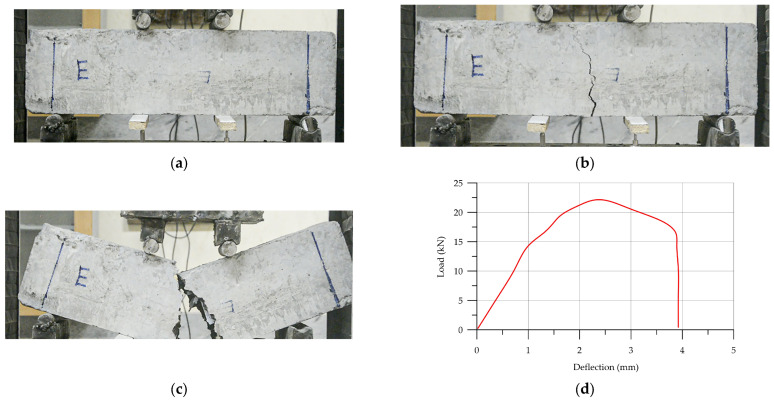
The load–deflection of WV-HPC: (**a**) initial sample of WV-HPC prism; (**b**) crack; (**c**) failure; (**d**) load–deflection diagram.

The specimens of concrete reinforced with Warren trusses with vertical racks demonstrated the results shown in Figure 15, and Figure 18a illustrates the failure process of the beam. Like with the failure process of WV-HPC, the cracking progress showed slow cracking and failure of the sample after 16 kN. After that, the concrete beam failed suddenly. Table 9 illustrates the details of load–deflection results.

**Figure 19:**
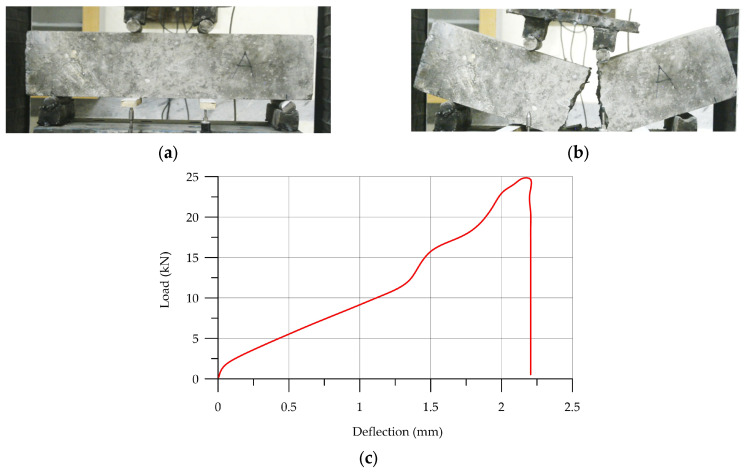
The load–deflection of HPC: (**a**) initial sample of HPC prism; (**b**) failure of HPC prism; (**c**) load–deflection diagram.

The results of the load–deflection diagram of the control sample are illustrated in Figure 19. According to Figure 19, the failure of the control sample was sudden and harsh. The load–deflection diagram is illustrated in Figure 19b. Table 10 shows the details of the flexural strength results of the control samples.

### 3.2. UHPC Reinforced with Hyperboloid Shell

#### 3.2.1. Compressive Strength Results

**Figure 20:**
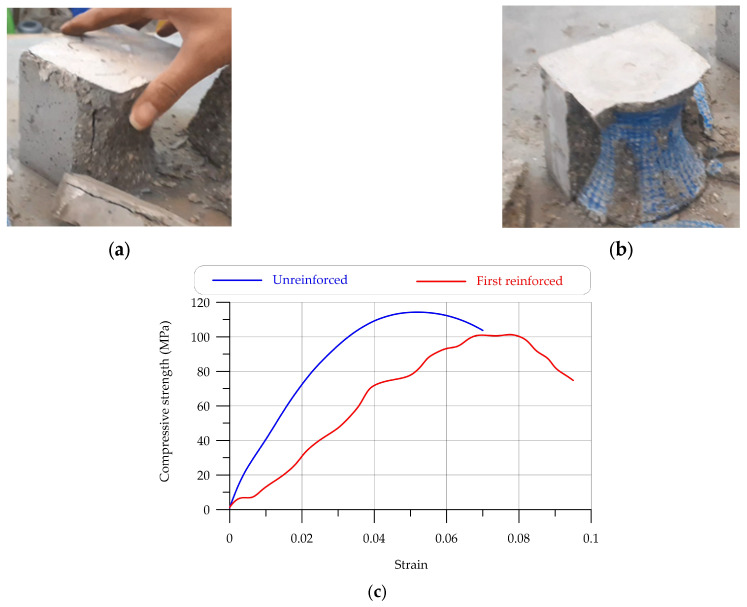
The compressive strength results of UHPC reinforced with hyperboloid shell: (**a**) unreinforced concrete sample; (**b**) reinforced concrete sample; (**c**) compressive load–deflection curves.

Two types of compressive cube were examined: one type was reinforced, and one type was the control sample. Figure 20 illustrates the compressive cube failure and the compressive stress–strain curve. Table 11 shows the compressive strength of all samples. According to Table 11, the average ultimate compressive strength was over 114 MPa for unreinforced concrete and 91 MPa for reinforced concrete. Based on the results, the strength of the reinforced concrete decreased by over 80% compared to unreinforced concrete.

#### 3.2.2. Tensile Strength Results

**Figure 21:**
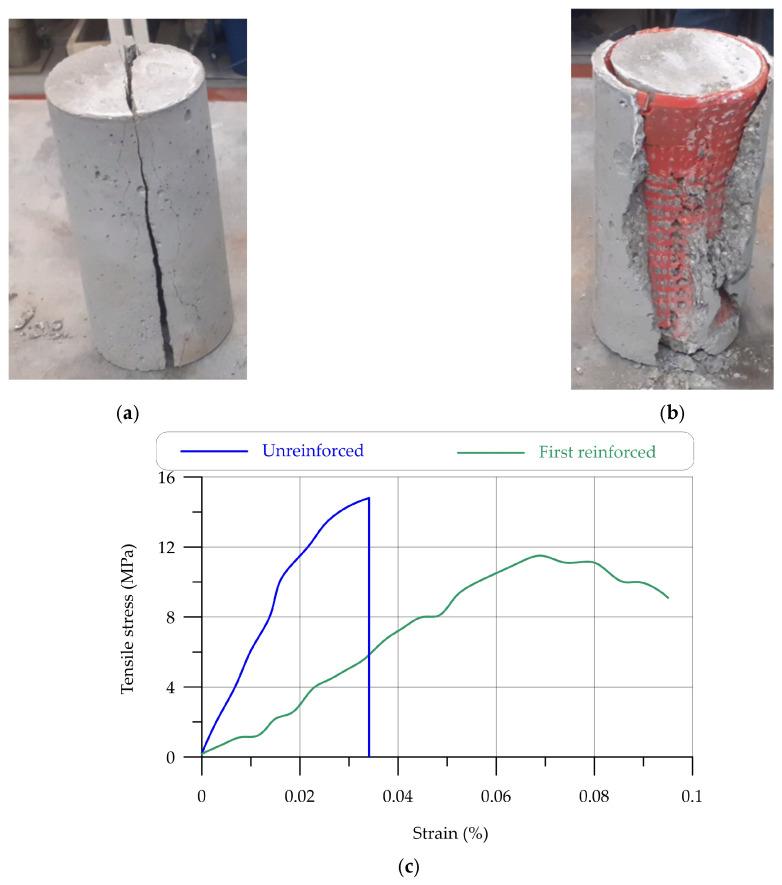
The tensile strength results of UHPC reinforced with hyperboloid shell: (**a**) unreinforced concrete sample; (**b**) reinforced concrete sample; (**c**) tensile load–deflection curves.

The tensile strength of the cylindrical sample demonstrates that the use of a hyperboloid shell structure in 3D-printing-reinforced concrete resulted in a partial failure of the sample, whereas the control sample completely broke under the applied load. Figure 21 illustrates the tensile stress–strain and failure of a cylindrical sample. Table 12 illustrates the tensile strength of reinforced and unreinforced UHPC.

#### 3.2.3. Three-Point Bending Test Results

**Figure 22:**
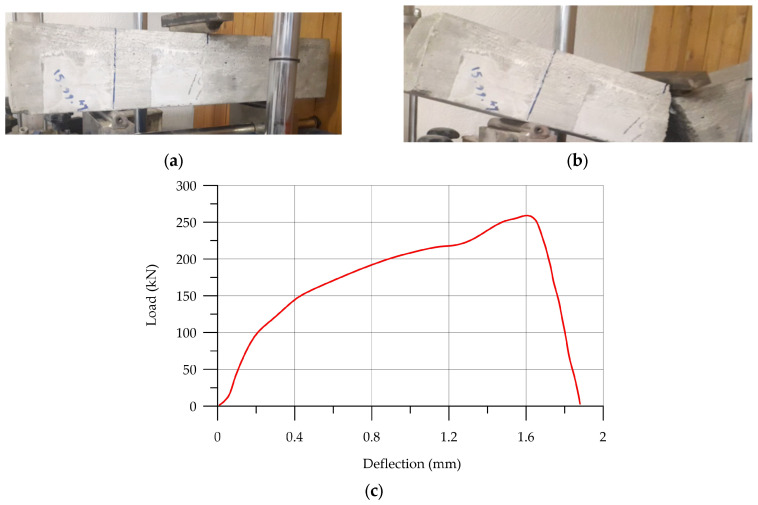
The flexural strength results of unreinforced UHPC: (**a**) initial UHPC sample; (**b**) failed sample; (**c**) load–deflection sample.

**Figure 23:**
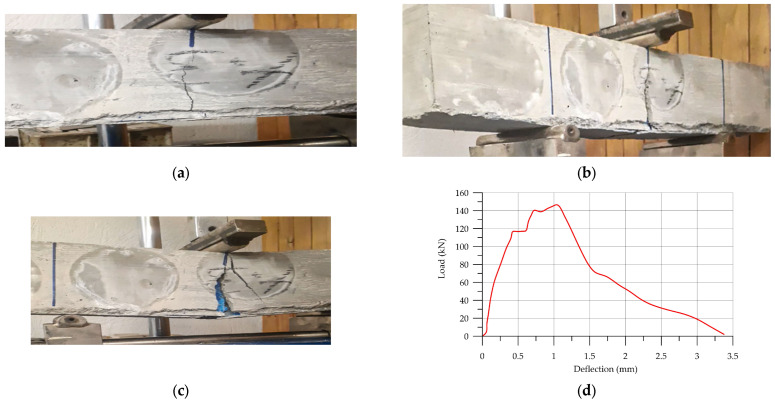
The flexural strength results of unreinforced UHPC: (**a**) initial crack; (**b**) crack in progress; (**c**) failed sample; (**b**) load–deflection sample.

Failure results and the stress–strain curve of unreinforced and reinforced UHPC in a three-point bending test are shown in Figure 22 and Figure 23, respectively. When subjected to a load of 247 kN (Figure 22), the unreinforced UHPC specimen exhibited a rapid and abrupt failure, while the reinforced UHPC did not fail after loading, as shown in Figure 23a. The first crack appeared when the specimen was loaded to 116 kN, and the crack progressed up to a loading force of 145 kN. Table 13 illustrates details of flexural strength.

## 4. Discussion

### 4.1. Differences between Two Types of Reinforcements

Based on the test results of HPC reinforced with trusses, it was found that the flexural strength increased by 26% compared to the control sample. The flexural strength of the Pratt-reinforced concrete, which was 3D-printed, decreased by over 34% compared to the control sample. According to research, the angle of inclination of the trusses exerts the strongest influence on their mechanical properties. For example, the optimal angles of inclination of truss elements for 3D printing are 50° and 60° [65]. Many investigations have been carried out on 3D-printed pyramidal trusses, concluding that an inclination angle of 65° demonstrates the maximum load capacity and the most favorable mechanical characteristics [66]. These results are similar to Warren’s truss inclination angle. Thus, the load capacity of W-HPC is greater than that of other samples.

According to the data presented in Table 14, the utilization of a hyperboloid shell structure as reinforcement for UHPC resulted in a decrease of over 41% in the flexural strength of the concrete, accompanied by an increase of over 45% in the maximum deflection. Therefore, hyperboloid shell structures can improve the deformation and ductility of UHPC samples under heavy loads.

The results of reinforced UHPC compressive tests show that when hyperboloid shell structures are used as a reinforcing structure, the sample did not fail completely, while the compressive strength decreased by more than 20% (Table 11, Figure 19). The tensile strength test results indicate that reinforcing the hyperboloid UHPC shell structure prevented specimen failure, although it resulted in a decrease in tensile strength (Table 12, Figure 20).

By comparing two 3D-printed samples, it was observed that the trusses produced by 3D printing were more useful and had more capability in bending loading than the hyperboloid shell structure. Importantly, when the hyperboloid shell structure was used as the reinforced shell structure, the maximum deflection was improved by as much as with the Warren truss. Hyperboloid shell structures can reinforce cubes to absorb energy; according to observations, the energy absorption of hyperboloid shell structure reinforcements under compressive strength is remarkable [67,68].

### 4.2. Differences between Current Study and Other Studies

Several studies have proved that when concrete is reinforced with 3D-printed structures, the strength of concrete is reduced. For example, Le et al. understood that when concrete was reinforced with 3D-printed PLA materials, the compressive and flexural strength reduced by over 17% and 45%, respectively [69]. Unlike other types of 3D-printed reinforcement, both types of 3D printing improved the ductility of the reinforced concrete beam, while in other studies, 3D-printed rebar decreased the deformation and displacement capacity of concrete [70,71]. Farina et al. [8] analyzed the reinforced concrete with 3D-printed rebar. According to their results, when concrete was reinforced with 3D-printed rebar, the behavior of flexural strength showed strain-softening. Additionally, the utilization of 3D-printing technology for rebars resulted in a decline in flexural strength and increased the fragility of the concrete. In contrast to the analysis conducted by Xu and Savija [72], it has been observed that the reinforcement of concrete with 3D-printed polymers can cause an increased deformation of concrete beams. Furthermore, Xu et al. [2] conducted an analysis of polymeric octet lattice structures in a 3D-printing-reinforced cement matrix. The findings indicate that the incorporation of 3D-printed polymer materials strengthened the cement matrix.

Many studies have understood that when 3D-printed reinforcements are added to concrete and cementitious materials, the mechanical properties improve. For example, Hao et al. [73] studied reinforced concrete with a 3D-printed polymer lattice, and the compressive strength increased by over 71%. In another example, when Chen et al. [74] reinforced UHPC with 3D-printed PLA, the compressive strength improved by over 8%. Rosewitz et al. [75] investigated the reinforced cement mortar beam with a different pattern of 3D-printed PLA material. In their results, the flexural strength improved when a special pattern was used to reinforce the cement matrix.

According to our results, when 3D-printed structures are added to a cement matrix or concrete, the mechanical properties of concrete can improve or decrease; the results of different studies illustrate that when 3D-printed elements with different geometries are added to cement matrix or concrete, the mechanical properties can improve. Therefore, the most important factor in the improvement of mechanical properties of concrete is the geometry of three-dimensional printing as a reinforcement. For example, auxetic materials are an effective structure to improve the mechanical properties of cement and concrete materials. Thus, when 3D-printed elements are added as reinforcements to cementitious materials, the mechanical behavior of concrete can be improved [76].

## 5. Conclusions

Three-dimensional printing technology can be used to reinforce concrete and cement materials. In this study, two types of 3D-printed geometries were selected to find the best structure for modifying cementitious structures. The current study was focused on HPC concrete reinforced with three-dimensionally printed trusses and UHPC concrete reinforced with a 3D-printed hyperboloid shell. Although the infill percentage of the spatial shell hyperboloid structure was 30 and the infill percentage of the 3D trusses was 45, the patterns are more important than the infill percentage. According to the results, HPC reinforced with trusses had better flexural strength than UHPC reinforced with a hyperboloid shell structure. In the results, the UHPC reinforced with the hyperboloid shell structure was capable of absorbing energy under compression loading. The current study concludes the following:

I. Hyperboloid shells and honeycomb structures can improve the energy absorption of reinforced concrete, although hyperboloid shell structures cannot improve the flexural strength of UHPC.
II. Warren and Howe types of trusses can increase the flexural strength of HPC as 3D-printed reinforcements. Adding these two types of 3D-printed reinforcements can improve the flexural strength by over 12% and 4.4%, respectively.
III. Research and studies show that the inclination angle of trusses is an important factor in increasing the mechanical properties and flexural strength. According to the results, the maximum mechanical properties of trusses are related to the inclination angle of 3D printing: 50° and 60°.
IV. The results of this study show that the main factor in improving the ductility and mechanical properties of concrete is related to the geometry of 3D-printed shapes.
V. This study suggests that further research into different types of hyperboloid shell structures to reinforce cementitious materials should be undertaken to find the positive or negative effect of hyperboloid geometry.

## Figures and Tables

**Table 1:** Details of PLA fabrication of hyperboloid and trusses.

Details	3D-Printed Trusses	3D-Printed Hyperboloid
Layer thickness (mm)	0.2	0.2
Printing speed (mm/s)	50	50
Infill percentage (%)	45	30
Extruder temperature (°C)	190	190
Bed temperature (°C)	60	60

**Table 2:** The properties of PLA materials [54].

Material	Ultimate Tensile Strength (MPa)	Yield Strength (MPa)	Maximum Strain (%)
PLA	57.16 ± 0.35	52.47 ± 0.35	2.35 ± 0.05

**Table 3:** Properties of silica fume [58,59].

Chemical Composition	Value (%)	
	Silica Fume	Cement
SiO_2_	90–92	19.52
Al_2_O_3_	0.68	4.81
Fe_2_O_3_	0.69	4.08
CaO	1.58	62.18
SO_3_	-	2.81
K_2_O	-	0.6
MgO	1.01	-
Na_2_O	0.61	-
K_2_O	1.23	-
C	0.98	-
S	0.23	-
L.O.I	-	1.67

**Table 4:** Mixture design of high-performance concrete.

Material	Cement (kg/m^3^)	Water (kg/m^3^)	Superplasticizer (kg/m^3^)	Sand (kg/m^3^)	Gravel (kg/m^3^)	Silica Fume (kg/m^3^)
HPC	500	187.5	12.5	585	1005	128

**Table 5:** Mixture design of ultra high-performance concrete.

Material	Cement (kg/m^3^)	Water (kg/m^3^)	Superplasticizer (kg/m^3^)	Fine Aggregate (kg/m^3^)	Gravel (kg/m^3^)	Silica Fume (kg/m^3^)
UHPC	420	105	12.6	945	635	65

**Table 6:** P-HPC load–deflection diagram results.

Sample	Maximum Load (kN)	First Crack Deflection (mm)	Maximum Deflection (mm)	Deflection Recorded by Left LVDT (mm)	Deflection Recorded by Right LVDT (mm)	Weight (kg)
P-HPC-1	15.9	3.04	5.03	2.68	4.03	45.3
P-HPC-2	15.5	3.09	5.11	2.73	4.09	45.1
P-HPC-3	16	2.93	4.92	2.61	3.96	45.3

**Table 7:** H-HPC load–deflection diagram results.

Sample	Maximum Load (kN)	First Crack Deflection (mm)	Maximum Deflection (mm)	Deflection Recorded by Left LVDT (mm)	Deflection Recorded by Right LVDT (mm)	Weight (kg)
H-HPC-1	25.5	3.19	4.61	0.78	1.25	45.5
H-HPC-2	24.3	3.12	4.42	0.70	1.21	45.3
H-HPC-3	26.2	3.28	4.78	0.78	1.30	45.4

**Table 8:** W-HPC load–deflection diagram results.

Sample	Maximum Load (kN)	First Crack Deflection (mm)	Maximum Deflection (mm)	Deflection Recorded by Left LVDT (mm)	Deflection Recorded by Right LVDT (mm)	Weight (kg)
W-HPC-1	27.9	2.64	4.45	3.52	2.85	44.6
W-HPC-2	27.6	2.86	4.58	3.63	3.01	44.6
W-HPC-3	28.1	2.41	4.23	3.44	2.63	44.5

**Table 9:** WV-HPC load–deflection diagram results.

Sample	Maximum Load (kN)	First Crack Deflection (mm)	Maximum Deflection (mm)	Deflection Recorded by Left LVDT (mm)	Deflection Recorded by Right LVDT (mm)	Weight (kg)
WV-HPC-1	31.9	2.42	3.94	2.56	2.88	43.5
WV-HPC-2	20.6	2.26	3.82	2.33	2.75	43.8
WV-HPC-3	22.3	2.59	4.03	2.74	3.02	43.6

**Table 10:** Control sample of HPC load–deflection diagram results.

Sample	Maximum Load (kN)	First Crack Deflection (mm)	Deflection Recorded by Left LVDT (mm)	Deflection Recorded by Right LVDT (mm)	Weight (kg)
HPC-1	24.4	2.21	0.25	0.21	46
HPC-2	24.1	2.41	0.28	0.26	45.8
HPC-3	24.6	2.10	0.22	0.18	46.1

**Table 11:** Compressive strength of UHPC reinforced with hyperboloid shell.

Samples	Unreinforced			Reinforced		
	First Sample	Second Sample	Third Sample	First Sample	Second Sample	Third Sample
Ultimate Strength (MPa)	113	117	110	99	82	93
Average Ultimate Strength (MPa)	114			91		

**Table 12:** Tensile strength of UHPC reinforced with hyperboloid shell.

Samples	Unreinforced			Reinforced		
	First Sample	Second Sample	Third Sample	First Sample	Second Sample	Third Sample
Ultimate Strength (MPa)	14.5	15.8	16.1	11.4	10.7	11.1
Average Ultimate Strength (MPa)	15.4			11.1		

**Table 13:** Flexural strength (three-point bending test) of UHPC reinforced with hyperboloid shell.

Samples	Unreinforced			Reinforced		
	First Sample	Second Sample	Third Sample	First Sample	Second Sample	Third Sample
Flexural Strength (kN)	38.1	40.5	42.6	33.1	35.2	38.8
Average Flexural Strength (kN)	40.4			35.7		

**Table 14:** Flexural strength of reinforced and unreinforced concrete.

Sample	Maximum Load (kN)	First Crack Deformation (mm)	Maximum Deflection (mm)
HPC	24.4	-	0.25
P-HPC	15.9	3.04	2.68
H-HPC	25.5	3.19	0.74
W-HPC	27.9	2.64	3.52
WV-HPC	21.9	2.42	2.56
Unreinforced UHPC	247.15	-	1.88
Reinforced UHPC	145.2	0.62	3.42

## Data Availability

The original contributions presented in the study are included in the article, further inquiries can be directed to the corresponding author.
